# First person – Emily Koller

**DOI:** 10.1242/dmm.049602

**Published:** 2022-05-16

## Abstract

First Person is a series of interviews with the first authors of a selection of papers published in Disease Models & Mechanisms, helping early-career researchers promote themselves alongside their papers. Emily Koller is first author on ‘ Temporal and spatially controlled APP transgene expression using Cre-dependent alleles’, published in DMM. Emily is a postdoctoral associate in the lab of Joanna Jankowsky, PhD at Baylor College of Medicine, Houston, TX, USA, investigating the impact of ageing in Alzheimer's disease, and is also interested in developing therapeutic interventions for diseases of the brain.


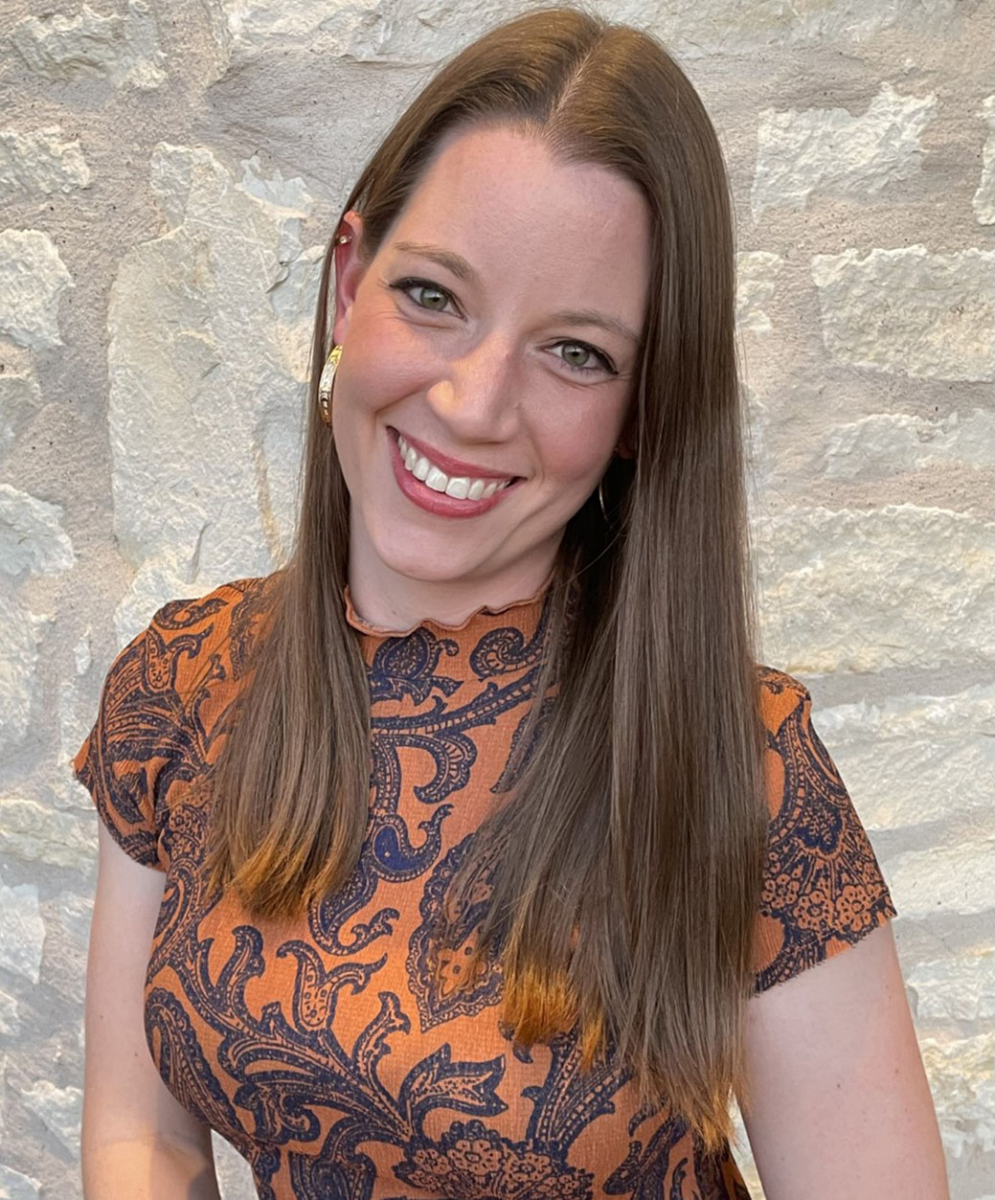


**Emily Koller**

**How would you explain the main findings of your paper to non-scientific family and friends?**

Alzheimer's disease is a devastating neurodegenerative disease characterized by progressive memory loss and impaired executive function and learning. The brain pathology underlying these clinical changes begins accumulating during midlife and progressively worsens over time, making age the greatest risk factor for developing Alzheimer's disease. Although numerous animal models have been created to study Alzheimer's disease, many of them begin accumulating Alzheimer's pathology early in life, creating a disconnect between time of disease onset in animal models and disease onset in humans. We sought to correct this temporal disconnect by developing new mouse models based on genetic tools to exert control over disease onset. Using these tools, we can choose when disease-causing transgenes such as the amyloid precursor protein (APP) become active in the brain, allowing us to generate mice with Alzheimer's pathology at any age. In addition, these systems allow for targeted expression, meaning we can choose which types of cells express APP in the brain. In summary, this study demonstrated effective strategies for achieving increased temporal and spatial control over transgene expression in Alzheimer's mouse models, and provided a new mouse line for the neurodegenerative disease research field.

“Since age imparts elevated risk for Alzheimer's disease, establishing additional mouse models […] is vital to evaluate why an aged brain is more vulnerable to Alzheimer's disease than a young brain.”

**What are the potential implications of these results for your field of research?**

A small percentage of Alzheimer's disease cases are caused by familial genetic mutations, while the majority of cases arise sporadically in the last decades of life. Since age imparts elevated risk for Alzheimer's disease, establishing additional mouse models with the option to induce disease onset during old age is vital to evaluate why an aged brain is more vulnerable to Alzheimer's disease than a young brain. Insight gained from Alzheimer's studies conducted in the context of an aged brain will also play a pivotal role in the development of therapeutics. There is no cure for Alzheimer's disease and only one disease-modifying treatment has been FDA approved: aduhelm/aducanumab. More research is needed to develop additional therapeutics and to determine effective treatment windows in disease progression. Alzheimer's disease models equipped with inducible pathology systems will spur this endeavor by allowing for studies dissecting the effect of aging in disease progression.

**What are the main advantages and drawbacks of the model system you have used as it relates to the disease you are investigating?**

The main advantages of using mouse models for research are: (1) mice are mammals and have physiology, anatomy and genetics that are similar to humans; (2) mice can be genetically manipulated for the study of diseases; and (3) mice have a high reproductive capacity and are a cost-efficient animal model for laboratory research. The disadvantage of using mice as a model is that they do not accurately mimic all phenotypes of human diseases. This means that some results from mouse studies may not be recapitulated in human studies.

The main advantages of the system we describe are that expression of pathogenic APP can be targeted to specific brain cells and timing of expression can be controlled through exogenous drug treatment. The disadvantage of our models is that Alzheimer's pathology develops slowly, which limits how late the transgene can be activated in order to see amyloid plaques within the remaining lifespan.

**Figure:**
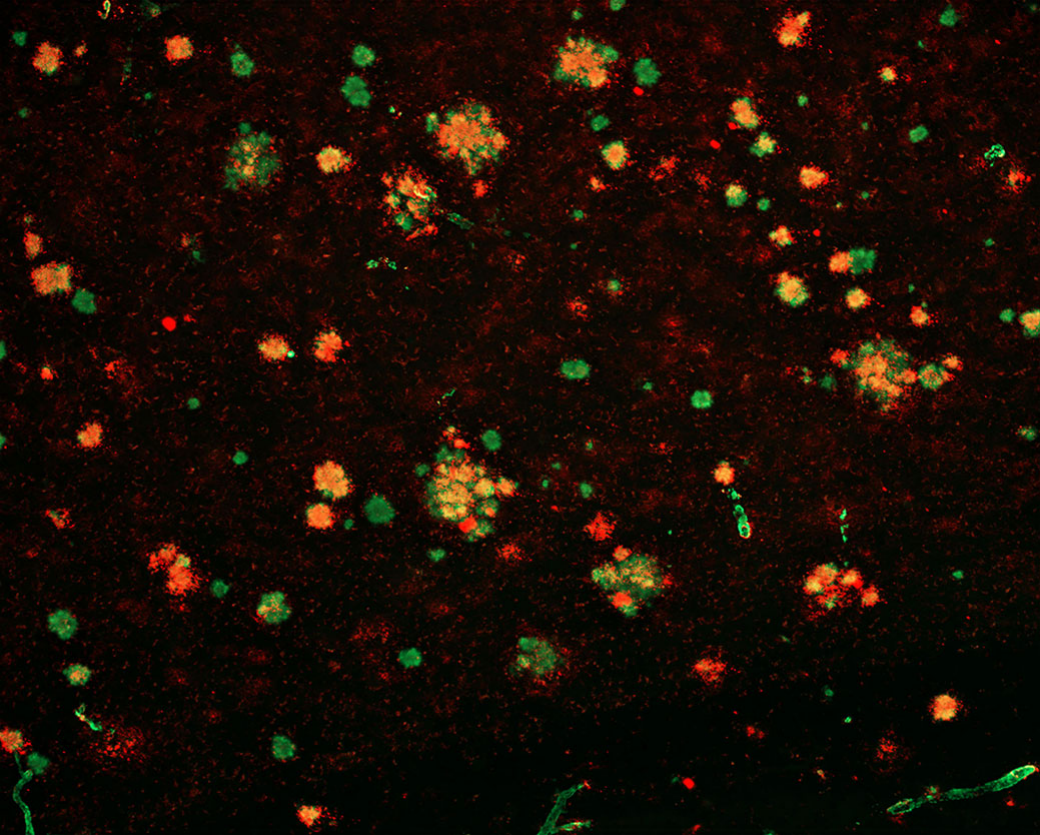
Image from the cortex of an Alzheimer's disease mouse brain depicting amyloid plaque pathology.

**What has surprised you the most while conducting your research?**

Two unexpected findings surprised us while conducting our research. First, we observed that some of the transgenic mouse combinations we tested resulted in animals that did not successfully express the transgene. In one example, a genetic combination that worked well on paper did not work at all in the mouse brain. Second, we found that transgene expression level was lower than anticipated and the time to develop pathology was longer than we had predicted. These observations highlight the importance of carefully characterizing new mouse lines because new transgene combinations can produce unexpected results.

**Describe what you think is the most significant challenge impacting your research at this time and how will this be addressed over the next 10 years?**

It is difficult to diagnose and treat Alzheimer's disease because the pathological proteins that cause the disease begin accumulating during midlife, while clinical symptoms present years later. One of the main challenges facing the field right now is finding reliable biomarkers to screen for Alzheimer's disease in individuals that are in early disease stages. Earlier diagnosis allows for the determination of the optimal treatment window for each individual; however, even once this challenge has been met, the continued development of disease modifying therapeutics is still necessary. Too many therapies that are effective in curing Alzheimer's disease in mice do not work when tested in clinical trials.

To address these challenges, the Alzheimer's research field has created mice with humanized genes, rendering the model system even more similar to a human with the hope that results will translate more successfully in clinical trials. Researchers are also studying the myriad of risk factors and co-morbidities – our research focuses on the risk imposed by age – that will introduce person-to-person variation when pursuing treatment.

**What changes do you think could improve the professional lives of early-career scientists?**

Career development and networking skills are vital for success in any field. In science, often the focus is on technical research skills, but it is also important to develop strong leadership, mentoring, collaboration and networking skills. We are not only scientists – we are also the next generation of leaders in our scientific field. Shifting the focus of career development and providing more opportunities to develop professional skills as well as technical skills will help propel early-career scientists into the next stage of their career journey.

**What's next for you?**

My long-term goal is to contribute to the design and development of disease-modifying therapies for the central nervous system.

## References

[DMM049602C1] Koller, E. J., Comstock, M., Bean, J. C., Escobedo, G., Park, K.-W. and Jankowsky, J. L. (2022). Temporal and spatially controlled APP transgene expression using Cre-dependent alleles. *Dis. Model. Mech.* 15, dmm049330. 10.1242/dmm.049330 35394029 PMC9118045

